# Post-Mastectomy Emergency Department Visits in Alberta: Understanding Patient Perspectives

**DOI:** 10.3390/curroncol33090537

**Published:** 2026-09-04

**Authors:** Emily M. Heath, Julia Chai, Susan Isherwood, Riley Martens Mulangu, Steven Langer, May Lynn Quan

**Affiliations:** 1 Department of Surgery, University of Calgary, Calgary, AB T2N 2T9, Canada; susan.isherwood@albertahealthservices.ca (S.I.);; 2 Cumming School of Medicine, University of Calgary, Calgary, AB T2N 2T9, Canada; chai@student.ubc.ca; 3 Department of Community Health Sciences, University of Calgary, Calgary, AB T2N 2T9, Canada

**Keywords:** breast, cancer, emergency, quality, surgery, satisfaction

## Abstract

Same-day surgery for mastectomy increased in Alberta from 1.7% to 73% in 2022, after implementation of a perioperative pathway in 2016. However, rates of unplanned visits to the emergency department (ED) remained >20%. Previous research explored reasons for this using administrative data but lacked patient-level data. Our study aims to explore patient-reported factors influencing unplanned ED visits after mastectomy. A survey study was conducted of patients who underwent mastectomy in Alberta between July 2021 and June 2022. Patients were identified from the Canadian Institute of Health Information database; chart review was performed to confirm ED visit details. Survey questions evaluated medical, socioeconomic, and psychologic domains, as well as patient-reported experiences of perioperative care. Of 556 patients who underwent mastectomy during the study period, 23% presented to the ED unplanned within 30 days. The survey was sent to 87 patients meeting inclusion criteria; 38% responded. Most patients presented on a weekend, stating the ED was the only choice available at the time. The most common patient concerns were related to infection (38%) and drain function (38%). Despite high postoperative ED visit rates, overall, 84% felt prepared for surgery, only 15% felt uncomfortable with drain management, and 78% of patients were satisfied with surgery. Difficulty accessing their surgical team postoperatively was reported as the main challenge. Future initiatives should focus on improved access to outpatient care and education on post-mastectomy emergencies.

## Simple Summary

After mastectomy surgery in Alberta, greater than 20% of patients visit the emergency department. Previous studies evaluating reasons for this used administrative data but lacked patient-level information. This study explores patient-reported experiences and reasons for unplanned emergency department visits after mastectomy using survey data. We found that 23% of patients who had a mastectomy between July 2021 and June 2022 in Alberta presented to the emergency department unplanned within 30 days of surgery. Overall, 84% felt prepared for surgery; only 15% felt uncomfortable with drain management. While 78% of patients were satisfied with surgery, difficulty accessing their surgical team postoperatively was reported as the main challenge. Patients reported concern for infection and drain issues as the most common reason for unplanned ED visit post-mastectomy. Patients identified problems with accessing surgical care after discharge. Future initiatives should focus on improved access to outpatient care and education on post-mastectomy emergencies.

## 1. Introduction

Same-day surgery for mastectomy increased in Alberta from 1.7% in 2011 to 73% in 2022, after implementation of a perioperative care pathway in 2016 [1]. This care pathway included a provincial steering committee and provided education and resources to providers, patients, and caregivers. Despite this, unplanned visits to the emergency department (ED) remained high at 22–27%, with less than 5% requiring readmission. The rates of unplanned ED visit post-mastectomy in Alberta are high compared to other jurisdictions, reporting rates of 3–12% [2,3,4]. The most common reasons for ED visits in these studies included pain, wound concerns, and infections.

A recent report of the Canadian Institute of Health Information’s (CIHI) National Ambulatory Care Reporting System (NACRS) data found that the number of unplanned ED visits increased by 1.1 million in 2022–2023 compared to the previous year [5]. In addition, there is a body of the literature identifying that a substantial number of unplanned ED visits are related to cancer care, including postoperative and chemotherapy-related concerns, which are often complex and not always emergent [6]. Breast cancer is among the top on this list. Readmission rates within 30 days of surgery are well established as an important quality metric [7,8,9,10,11].

We previously explored factors associated with post-mastectomy ED visits using administrative data in a provincial, population-based study. Rates of unplanned ED visits after mastectomy did not change with implementation of a perioperative pathway [12], despite focused teaching about common postoperative concerns and being provided contact information for assessment out of the ED [1]. We identified concerns regarding incisions and infection as the most common reason for ED visits [12]. The study, however, lacked patient-reported data.

Unplanned ED visits after recent discharge or surgery are now gaining more attention as important quality care indicators [11,13,14,15,16,17], suggesting preventable encounters, such as those that could be managed in a non-urgent setting or offset by delivery of improved quality care [13]. Efforts to describe and understand unplanned and avoidable emergency visits are warranted to ensure safe, streamlined, and high-quality care for breast cancer patients.

The aim of this study is to explore factors influencing unplanned ED visits after mastectomy in Alberta using patient-reported data. Objectives include identifying and describing patients with unplanned ED visits within 30 days of mastectomy in Alberta in a post-perioperative pathway implementation cohort. We also aim to explore characteristics of and reasons for unplanned ED visits after mastectomy in the same cohort from the patient perspective.

## 2. Materials and Methods

### 2.1. Patient Population

The study population was identified from the CIHI discharge abstract database (DAD) and NACRS, which capture health system encounters, including procedures and ED visits. We identified patients who underwent mastectomy in Alberta between 1 July 2021 and 30 June 2022 and had an unplanned ED visit within the following 30 days. A chart review was performed to confirm surgery and ED visit details. Patients were excluded if their ED presentation was unrelated to surgery, if they were deceased at the time of the study, or if surgery was for reasons other than breast cancer treatment or was prophylactic, as these patients would not have been included in the cancer perioperative pathway.

### 2.2. Study Design

This was a survey study to explore characteristics of unplanned ED visits from the patient perspective. The survey was developed to evaluate patient experiences in the pre-, peri-, and postoperative phases of care, as well as ED visit care. We included 40 questions evaluating medical, socioeconomic, and psychologic domains. Preoperative care focused on educational resources received, whether they were read and understood, and patient satisfaction with preoperative education. Perioperative care focused on type of treatment and surgery type, prescriptions received, and any additional teaching prior to hospital discharge. Postoperative care focused on satisfaction, experiences of pain, functionality, and quality of life within 30 days of surgery, and suggestions for improvement in education. Finally, we asked about ED experience, medical and social reasons that influenced the decision to present to ED, and what other resources were considered. The survey was tested on a pilot population that had a mastectomy in Alberta in June 2021 or July 2022 (months before and after our study cohort) and had an unplanned ED visit within 30 days of surgery. Feedback included minor changes to survey organization and was incorporated into the final version used in the study.

### 2.3. Survey Methods

All patients meeting inclusion criteria after chart review were invited to participate. The survey was sent via email to patients who had an email address available and by mail to those who did not. Follow-up calls were completed for patients who did not respond within three weeks of survey administration.

Descriptive statistics were used to characterize the cohort and survey results. Free text comments provided by patients were reviewed and categorized by phase of care and type of comment, i.e., description of care, feedback, or suggestion for improvement. As a quality improvement initiative with minimal risk, this initiative did not require approval from our institution’s health research ethics board as per the “A Projects Ethics Community Consensus Initiative (ARECCI) Ethics Screening Tool” https://arecci.albertainnovates.ca/ (accessed on 5 October 2022).

## 3. Results

### 3.1. Patient and ED Visit Characteristics

**Figure 1:**
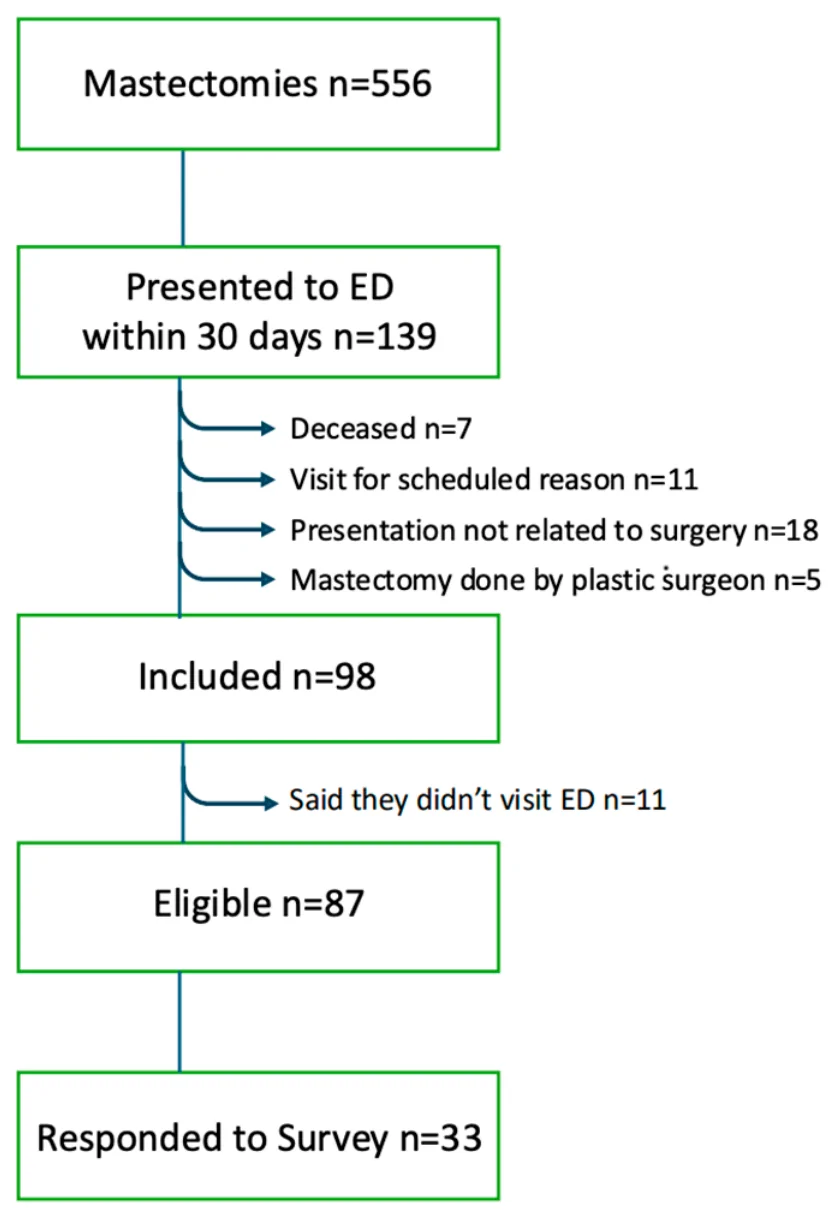
Flow diagram showing patients’ eligibility and participation in the study survey.

Of 556 patients who underwent mastectomy during the study period, 128 (23%) presented to the ED unplanned within 30 days (Figure 1). Ninety-eight patients met inclusion criteria and were contacted; however, 11 stated that they did not recall visiting the ED within 30 days of surgery. The survey was thus sent to 87 patients, of which 38% responded. Characteristics of patients who responded to the survey are displayed in Table 1. The average age was 56. Most patients had at least one comorbidity and history of prior surgery of any sort, including those unrelated to breast cancer diagnosis.

Of those who could recall, most patients (80%) presented to the ED on a Friday, Saturday, or Sunday (herein classified as a weekend), of which 41% stated the ED was the only choice available at the time. Comparatively, 22% reported being told to go, and 16% felt the ED was the best place for their problem. Of patients who were told to go to the ED, they commented that they were told by a family member, neighbor, nurse, family physician, or surgeon’s office. Seven patients reported attempting to contact their surgeon’s office prior to presenting to the ED; only two were successful, and both reported being told by the office to go to the ED. Reasons for not contacting the surgeon’s office include the office being closed, it being a long weekend, or being told by someone else to present to the ED prior to contacting the surgeon.

**Figure 2:**
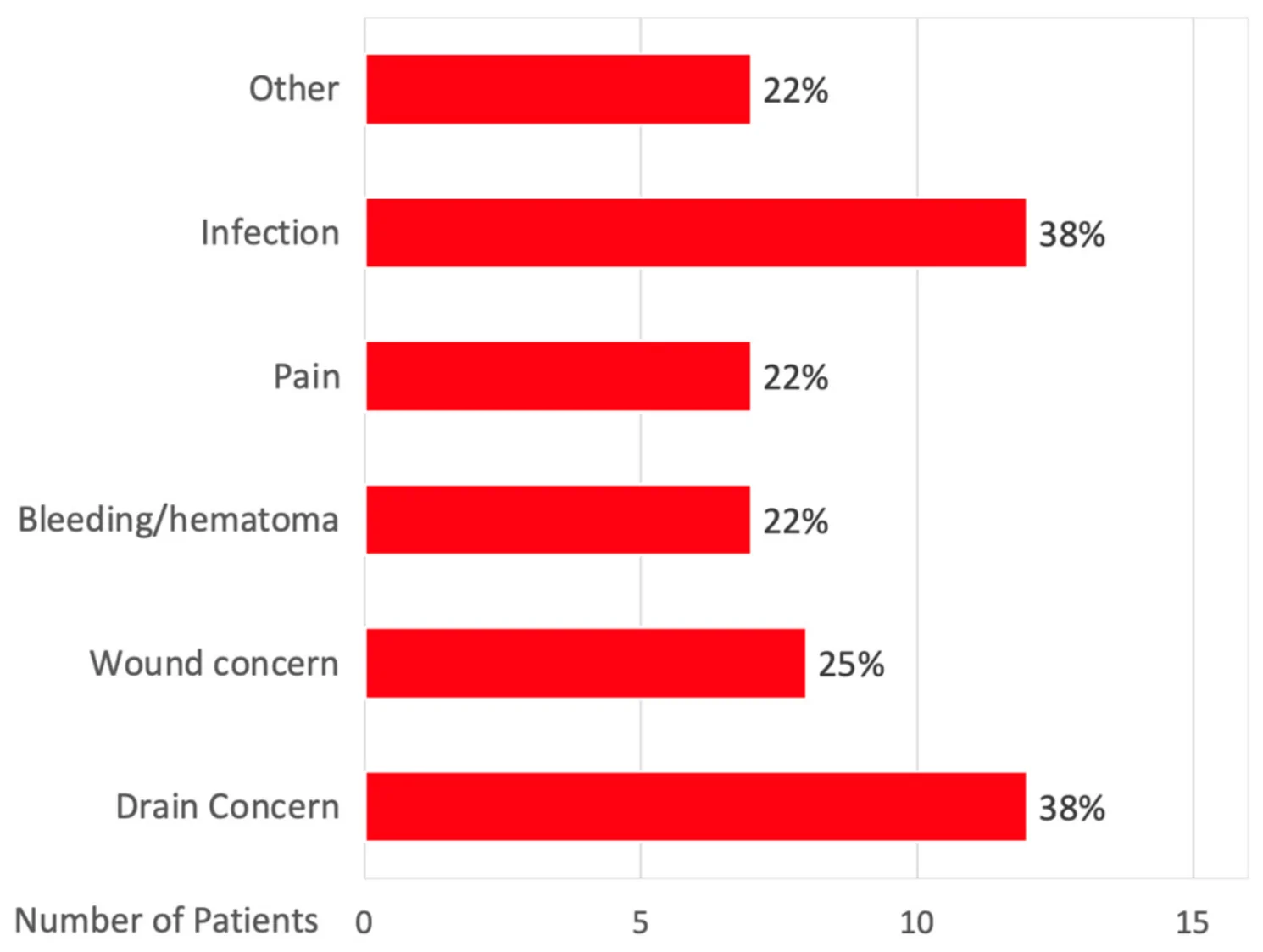
Patient-reported reasons for seeking medical care after mastectomy.

**Figure 3:**
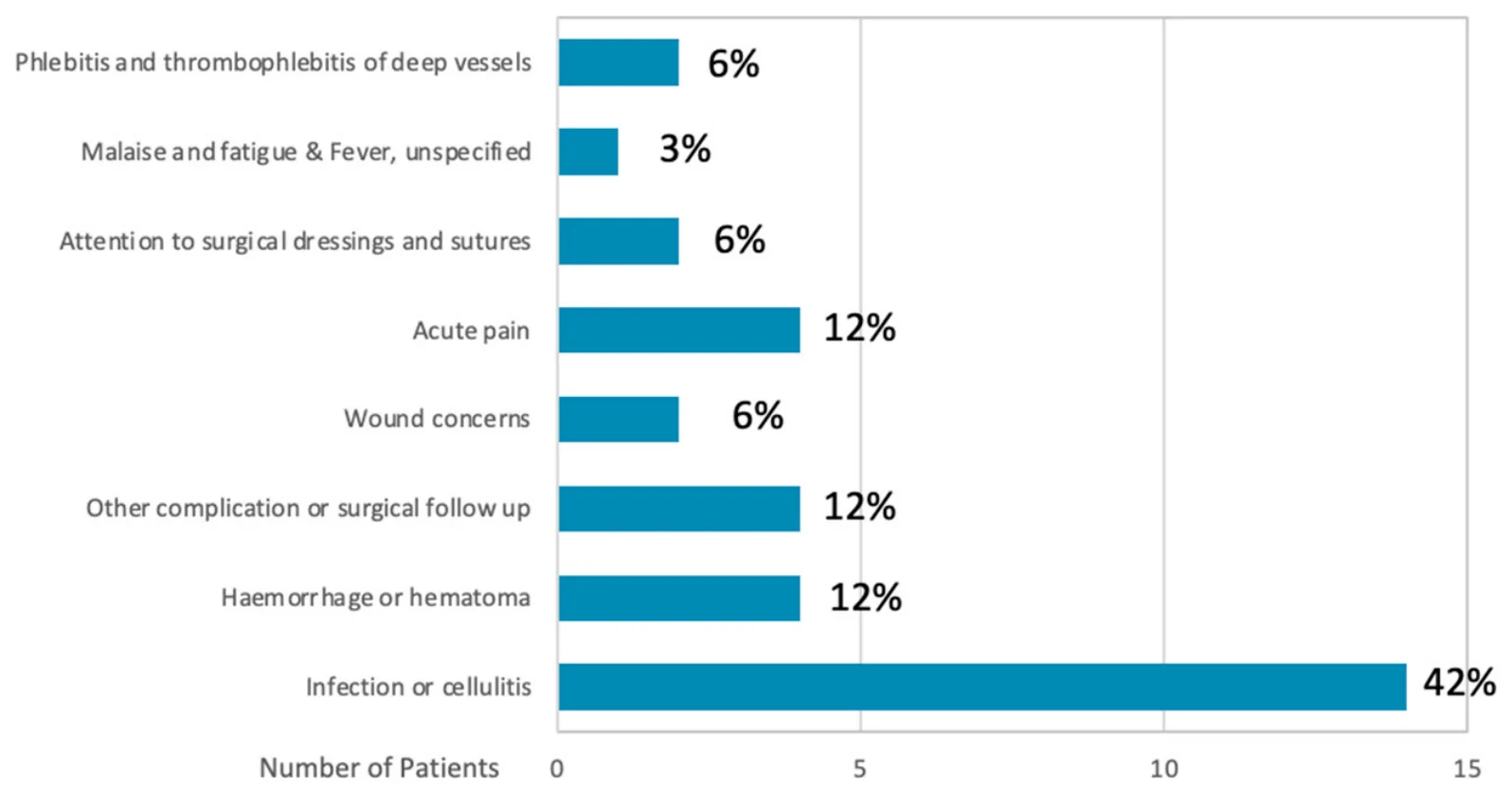
Administrative data regarding reasons for an ED visit after mastectomy.

Patients presented to the ED, on average, 9.8 days following surgery based on administrative data (range 0–26 days). Patients recall being seen by an ED physician (89%), nurse (40%), and/or surgeon (28%). The most common patient concerns prompting an ED visit were infection (38%) and drain function (38%) (Figure 2). Fifteen patients presented to the ED within 7 days of surgery, with the most common reasons being drain concerns (47%) or bleeding (27%). Eighteen patients presented to the ED 8–30 days after surgery, with the most common reasons being infection (50%) and wound concerns (33%). Administrative data citing reasons for the ED visit are shown in Figure 3. Readmission and reoperation rates were 24% and 18%, respectively. Surgery took place on a Monday in 15% of patients, Tuesday in 27%, Wednesday in 15%, Thursday in 27%, and Friday in 15%, based on the administrative data. The location of surgery was in a major city, such as Calgary or Edmonton, in 22 patients and in a smaller city, with a population less than 150,000, in 11 patients.

### 3.2. Preoperative Care

**Figure 4:**
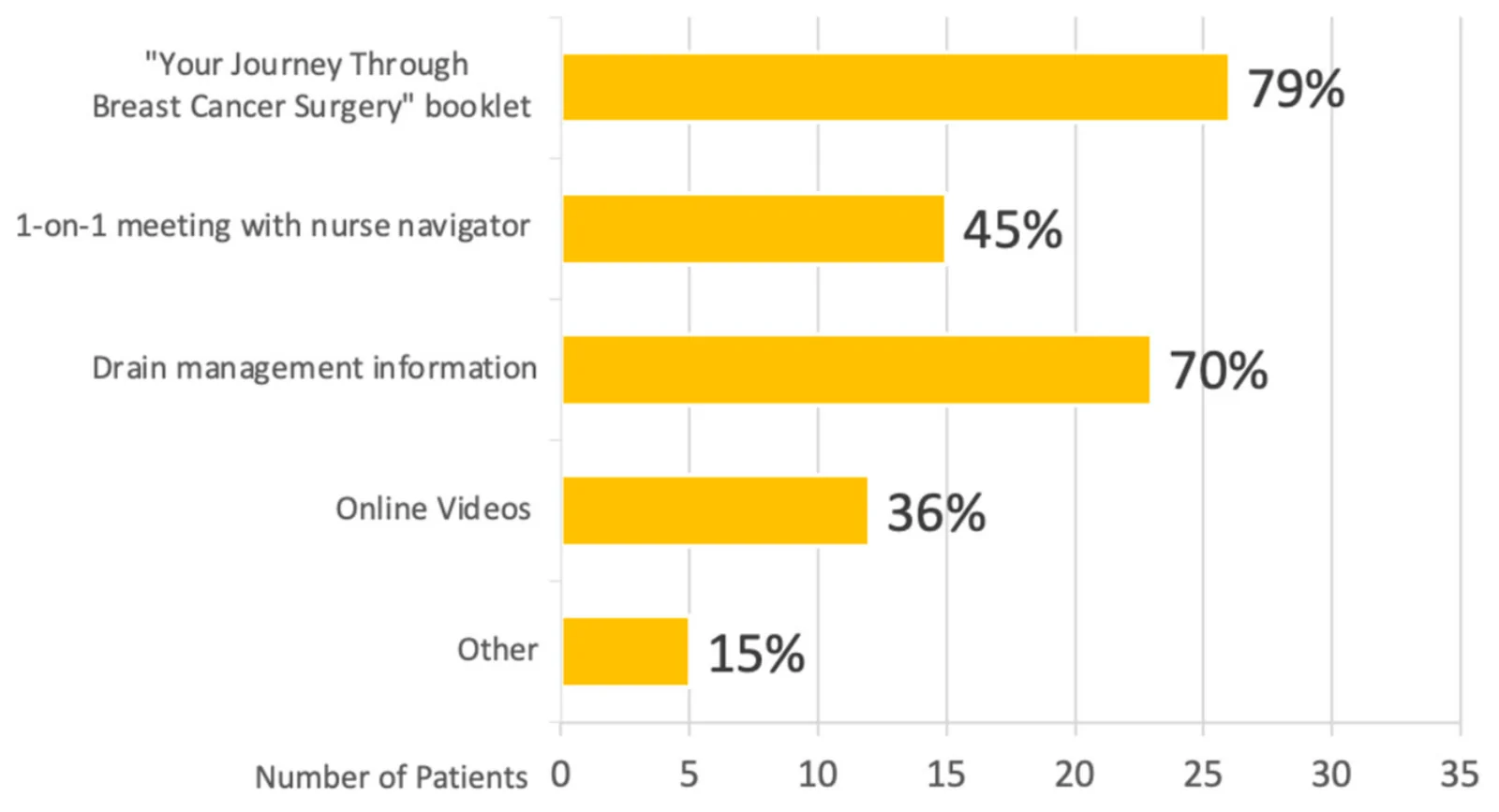
Preoperative resources used by patients prior to mastectomy.

**Figure 5:**
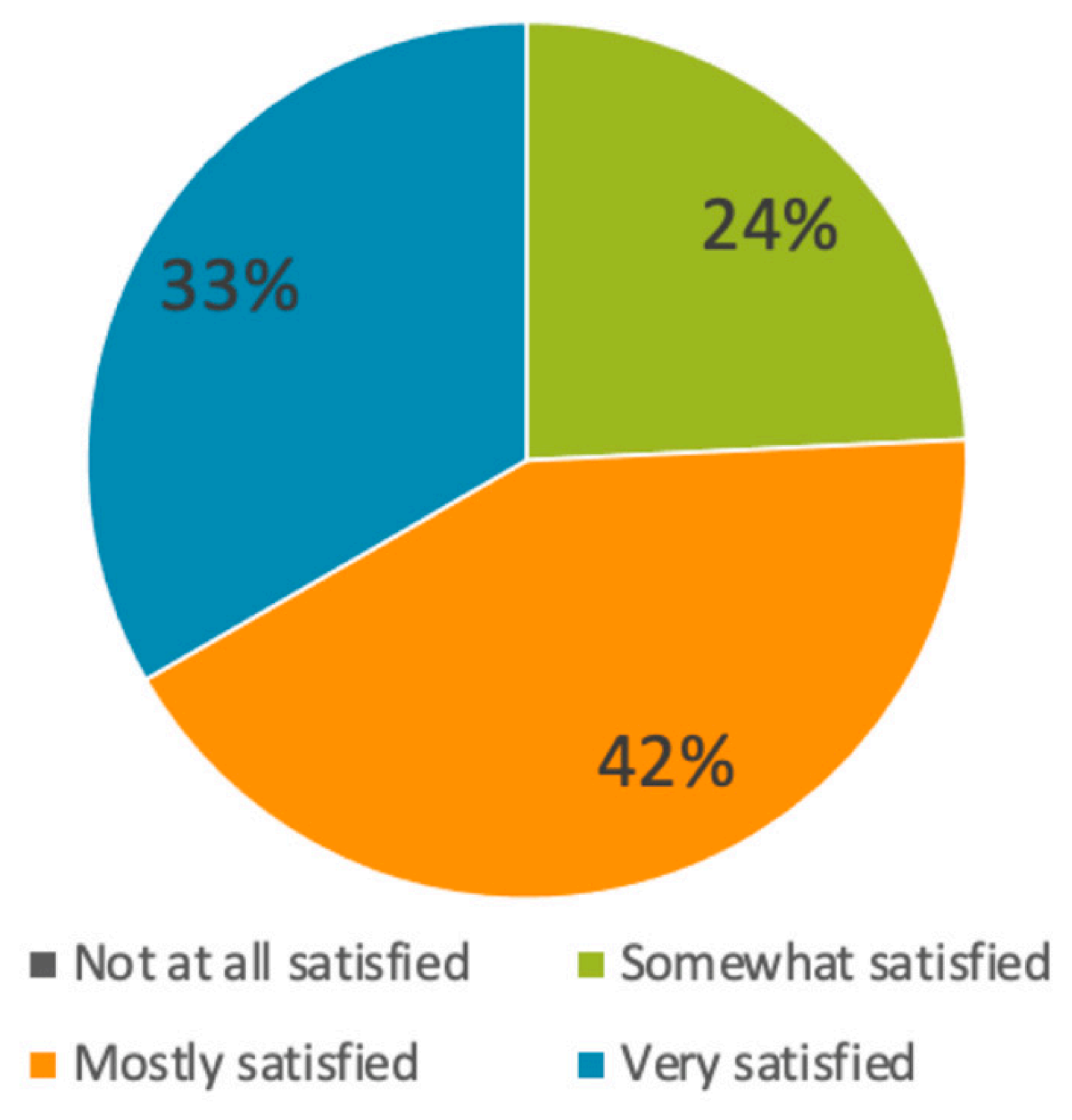
Patient-reported satisfaction with preoperative care and resources.

Figure 4 shows preoperative resources that patients received prior to their surgery. Most patients reported that they were satisfied with their preoperative care, and none were unsatisfied (Figure 5). About 25% of patients said there were aspects that could have been improved. Patients commented “drain demonstration and actual models with realistic photographs would have been helpful”, “more education and information on scar tissue would have been welcome”, and “I would have liked peer support and to hear other patient experiences”.

### 3.3. Perioperative Care

**Figure 6:**
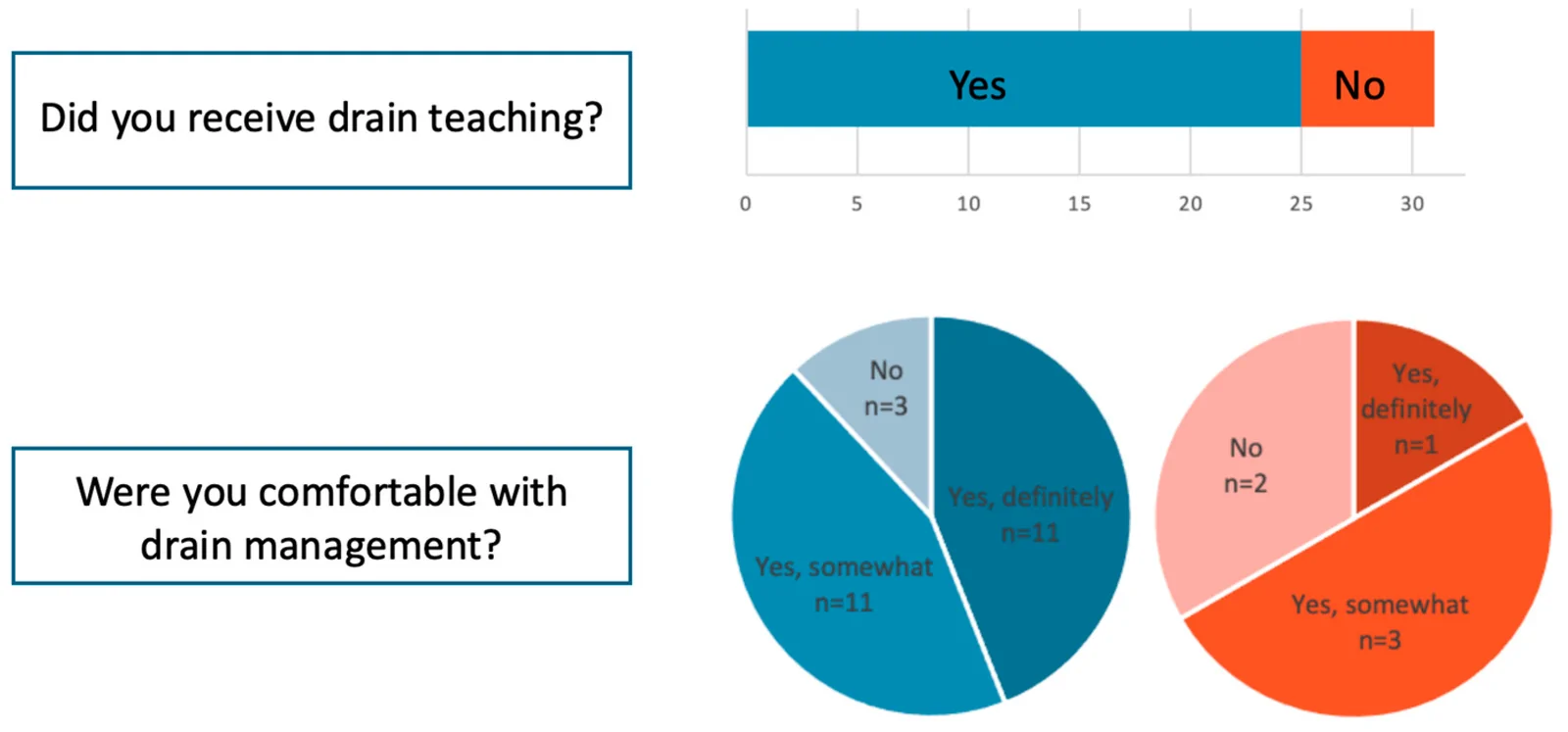
Patient-reported drain teaching and comfort with drain management after mastectomy.

Characteristics of medical and surgical treatments recalled by patients are displayed in Table 2. Patients underwent a range of treatments in addition to mastectomy, including neoadjuvant chemotherapy, lymph node procedures, and reconstruction. Of the patients who were readmitted, two had undergone mastectomy and sentinel lymph node biopsy, one had a modified radical mastectomy, four had mastectomy alone, and one had immediate reconstruction with an implant. Of the patients who had a reoperation, three had mastectomy alone, and three had mastectomy with immediate reconstruction (two with implants, one autologous). Reasons for reoperation based on the chart review included wound debridement in the context of reconstruction in three patients, wound debridement without reconstruction in one, and evacuation of hematoma in two. Most patients had one to two surgical drains, although 24% had three or more drains. Most patients received perioperative drain teaching; however, a small proportion of patients did not recall receiving drain teaching at all. Despite this, most patients recalled being comfortable with managing their drain before they left the hospital (Figure 6). Regardless of comfort, 74% of patients had someone at home to assist with drain management.

### 3.4. Postoperative Care

Overall, 84% felt prepared for surgery, and 78% of patients were mostly or very satisfied with surgical care overall. Difficulty accessing their surgical team postoperatively was reported as the main concern, with patient’s commenting “I had home care but was not impressed with them”, “frequent follow-ups with my surgeon were the most helpful”, “I had questions but could never get hold of my surgeon”, “would have liked a short-term follow up to stay connected”, and “I had a simple problem. Would have been nice to go to a (breast) outpatient clinic rather than wait in the ED”.

## 4. Discussion

Following implementation of a same-day surgery pathway designed to improve the perioperative care experience after mastectomy, rates of unplanned postoperative ED visits remained high in Alberta at 25%. In our study, although rates of reoperation and readmission were higher than previously reported, we found that most patients presented to the ED on a weekend (Friday, Saturday, or Sunday) and felt that they had no other options for seeking care. Concerns for infection and drain function were the main reasons for seeking care following mastectomy. Despite most patients feeling prepared for surgery and satisfied with their perioperative care, they identified better access to outpatient care and different methods of education delivery as areas for potential improvement.

### 4.1. Patient Cohort

Compared to the previous, larger population of patients studied by our group, this study cohort represents a smaller, more recent cohort of patients with unplanned ED visits within 30 days of mastectomy [12]. They are otherwise of similar average age, comorbidities, and socioeconomic status as evaluated by household income and education level. The mean age of our cohort is also similar to that found in other studies [2,3]. Comparison of comorbidities and socioeconomic status is difficult given the variety of comorbidity scores and scales used, as well as differences in currency and geographic location.

Patients in our cohort described a range of treatments received. While rates of sentinel lymph node biopsy and neoadjuvant chemotherapy were comparable to those in the patient population described by Vuong et al., fewer of our patients reported undergoing axillary lymph node dissection, representing 12% compared to 42%. Our rates of axillary dissection may be representative of the general trend toward less invasive axillary staging [18,19,20] or may be related to recall bias. About 24% of patients still recall having three or more surgical drains due to undergoing axillary lymph node dissection (*n* = 4) or autologous reconstruction (*n* = 3). The number of drains is notable given that drain concerns are a major driver of ED visits in our patient cohort, as discussed below.

### 4.2. Reasons for Unplanned ED Visits

In this study, the most common reasons patients reported for presenting unplanned to the ED were concerns regarding infection and drain function, each representing 38%. These proportions are at least twofold higher than those identified in our previous study using population-based data, where incision-related concerns were the most common reason for presentation at 17.4%, followed by skin infections at 14.4% [12]. Drain concerns represented only 11.1% of presentations in that study. Notably, that study used administrative data to obtain visit reasons, and thus, it is possible that some infections and drain concerns are captured within the incision-related concern category, leading to an underestimate of these reasons. It is also possible that patient-reported reasons for ED visits were different than the problem diagnosed by medical professionals in the ED; in fact, only 6% of patients in this study were diagnosed with wound concerns according to the administrative data. A total of 18% were diagnosed with “other concerns” or “attention to surgical dressings”, but drain concerns were not captured by the administrative data. The number of patients concerned about and diagnosed with infection according to the administrative data is similar in this study. However, reasons for unplanned ED visits after mastectomy vary across other published data as well. Existing literature suggests that pain is a common reason for ED visits after mastectomy [2] and after ambulatory surgery in general [21], and that consistent analgesia administration and prescriptions may decrease the likelihood of a patient seeking emergency care [3]. In our cohort, however, pain was not a driving factor for return to the ED. This likely reflects the efforts in our perioperative pathway, which include education on proper use of non-opioid analgesia and early ambulation and range of motion exercises for the affected arm.

When examining reasons for return to care in elderly patients following breast surgery, Westley et al. identified infection as the main reason; however, this included all non-breast-related infections as well; only 11% of patients actually returned with concern for skin and soft tissue-related infection [4]. Drain concerns were not listed as a reason for return to care.

Overall, these data suggest that reasons for unplanned ED visits after mastectomy vary across different settings and age groups. The lack of patients presenting with pain in our cohort may suggest that our perioperative pathway is successful in educating patients about postoperative pain management. However, the high proportion of patients presenting with infection and drain concerns may indicate that education is lacking in these areas, or that they had more trouble seeking non-emergency-level care for these issues. Some patients in our cohort did in fact identify drain teaching and education on wound scar tissue as areas that could be improved in the preoperative education.

### 4.3. Timing and Logistics of ED Presentation

Regardless of the variation across the literature regarding reasons for ED visits, many of the post-mastectomy concerns, such as drain concerns or wound concerns, are often non-urgent in nature and potentially manageable in alternative ambulatory settings. In this study, we sought to understand if patients truly felt that their issues required emergency care, in which case education may be lacking, or if access to non-urgent care was the bigger issue. Only 16% of patients in our study presented to the ED because they felt it was the best place for their problem, whereas 41% chose the ED because it was the only place available at the time they sought care. Interestingly, many patients presented to the ED on a weekend. This is one of the first studies, to the best of our knowledge, to consider day of the week in relation to post-mastectomy unplanned ED visits. A previous study considering ED visits within three days of hospital admission actually showed an opposite effect where re-attendance rates were lower from Friday to Sunday [22]. The authors speculated that this may be related to lower stress levels on weekends, which may reduce symptoms of illness or increase the likelihood of availability of family members on weekends to care for patients at home. When considering general ED visits, however, other data suggest there is an increased number of ED visits from Friday to Sunday as well as on public holidays, with a higher proportion of less urgent cases on these days [23]. In our patient cohort, weekend visits may have been a result of inability to access non-urgent care on weekends, with many surgeons’ and general practitioners’ offices closed. This is further supported by patient comments that they had trouble connecting with their surgeon or accessing an outpatient breast clinic for their problems, or that they could not contact their surgeon’s office due to the timing of their concerns being on a weekend or after hours. The surgical schedule did not seem to influence the timing of presentation, as there was a similar proportion of surgeries that occurred earlier in the week compared to later. The average number of days between surgery and ED presentation was also greater than 1 week with a large range, indicating that surgical scheduling has minimal effect on the timing of presentation.

There was a proportion of patients who stated they presented to the ED because they were told to go by a family member, neighbor, nurse, or other health care professional. This may, in part, be attributable to a phenomenon postulated in our prior work, where patients were instructed to present to the ED after calling a generic “811” provincial nursing advice line [12], which was not engaged in the perioperative pathway initiative [1] and may have a lower threshold to recommend seeking care in the ED.

Together, these data support an opportunity to improve access to outpatient care, especially after hours. This may include after-hours home care visits or virtual follow-ups for patients deemed at high risk of struggling with postoperative care or displaying high levels of anxiety surrounding surgery. Select patients may also benefit from routine, early postoperative check-ins from our clinic nurse navigators, particularly on Thursdays or Fridays, to provide reassurance and education on issues that may arise in the early postoperative phase. Research in orthopedic surgery supports similar initiatives, including phone calls, secure messaging, and early clinic visits customized around patients’ risk profiles [24], with evidence that implementation of a phone consultation service and increased use of outpatient clinics reduced ED utilization following joint arthroplasty [25,26].

### 4.4. Readmission and Reoperation Rates

Notably, our study cohort reported readmission and reoperation rates of 24% and 18%, respectively. These are unexpectedly high compared to other published data, which demonstrate readmission rates following breast surgery ranging from 0.9% to 6% [1,3,8,12,27,28] and reoperation rates ranging from 1.2% to 4% [12,29]. One contribution to this discrepancy may be an unclear definition of “reoperation”, as interpreted by patients. It is possible that some infections required debridement in the ED or clinic, or that reoperations were for reasons unrelated to the ED visit.

Another likely contributor to our high reported reoperation and readmission rates is survey bias, in that patients were more likely to fill out the survey if something significant resulted from their unplanned ED visit. This bias may also help understand the reported reasons for unplanned ED visits, which differ from those reported in other studies, as mentioned above. This is supported by additional literature that identified infection as one of the most common reasons for reoperation [29]. Other literature also found that patients who underwent axillary dissection were also more likely to have reoperation [29]. While these patients represented a significant portion of our survey responders, we did not see the same trend. Instead, none of our patients requiring reoperation had an axillary lymph node dissection; however, 50% had concurrent reconstruction at the time of their mastectomy. Vuong et al. identified similarly high reoperation and readmission rates of 24% and 17%, respectively, in patients presenting to the ED within one week of mastectomy [3].

### 4.5. Limitations

There are a few notable limitations to our study. As mentioned above, survey bias likely significantly contributes to our results, including the high reoperation and readmission rates as well as the high rates of axillary dissection. For this reason, our results should be interpreted mainly for the patient feedback and quality improvement rather than the proportional outcomes by surgery type. In addition, our exclusion criteria limit our analyses to ED presentations related to surgery; however, this may result in an underestimate of unplanned ED visit rates as there may be a lack of awareness of what constitutes a symptom related to surgery. For example, patients presenting with respiratory symptoms, venous thromboembolism, or gastrointestinal symptoms may be related to general anesthesia and intubation, immobility in the perioperative phase, or preoperative antibiotics. We also acknowledge the small sample size included in our study. Finally, our data are limited by recall bias as patients may not be able to accurately recall the details of their medical/surgical treatments or may not remember the specific teaching materials provided to them up to a year prior.

## 5. Conclusions

Our study found that infection and drain concerns were the most common patient-reported reasons for unplanned ED visits after mastectomy, and infection was the most commonly diagnosed problem in the ED according to administrative data. Overall, patients were satisfied with education and surgical care but identified problems with accessing medical/surgical care after discharge. Discordance between patient satisfaction and education with high ED visit rates highlights access to expert care as a main gap in postoperative patient care. Future initiatives should focus on improved access to outpatient care, short-term follow-up for high-risk patients, and education on post-mastectomy emergencies.

## Figures and Tables

**Table 1:** Self-reported characteristics of patients who had an unplanned ED visit within 30 days of mastectomy and responded to the study survey.

Characteristic	Category	Number (%)
Age	Average number of years	56 (32–87)
Comorbidities	Asthma	6 (18%)
	Autoimmune disorder	6 (18%)
	Diabetes	0 (0%)
	COPD	7 (21%)
	Heart condition	2 (6%)
	Hypertension	7 (21%)
	None	10 (30%)
	Other	8 (24%)
	2+ comorbidities	9 (27%)
Substance Use	Smoking	1 (3%)
	Alcohol use (of any amount)	10 (30%)
	Recreational drug use (incl. Marijuana)	1 (3%)
	None of the above	21 (64%)
Prior to Surgery	No prior surgery	5 (15%)
	1 surgery	7 (21%)
	2 surgeries	6 (18%)
	3+ surgeries	15 (45%)
Education Level	Did not graduate high school	3 (9%)
	High school graduate	4 (12%)
	2–4-year post-secondary degree	20 (60%)
	More than 4-year post-secondary	5 (15%)
	Prefer not to answer	1 (3%)
Annual Household Income	CAD 20–60,000	5 (15%)
	CAD 60–80,000	3 (9%)
	CAD 80–100,000	6 (18%)
	>CAD 100,000	11 (33%)
	Prefer not to answer	8 (24%)
Living With	Alone	7 (21%)
	Spouse/partner	26 (79%)
	Child(ren)	13 (39%)
	Parent(s)	1 (3%)

**Table 2:** Medical and surgical treatments recalled by patients.

Characteristic	Category	Number (%)
Type of Treatment	Neoadjuvant chemotherapy	8 (24%)
	Sentinel lymph node biopsy	13 (39%)
	Axillary dissection	4 (12%)
	Concurrent reconstruction	8 (24%)
	Mastectomy alone	9 (27%)
Number of Operative Drains	0	1 (3%)
	1	9 (27%)
	2	15 (45%)
	3+	8 (24%)

## Data Availability

The datasets presented in this article are not readily available due to privacy and ethical restrictions to protect the identity of participating patients.

## Abbreviations

The following abbreviations are used in this manuscript:

ED	Emergency Department
CIHI	Canadian Institute of Health Information
NACRS	National Ambulatory Care Reporting System
DAD	Discharge Abstract Database
